# Progress in Neurology

**Published:** 1903-08-08

**Authors:** 


### PROGRESS IN NEUROLOGY.

**Epilepsy.**—Turner[1] has analysed the records of the National Hospital for the Paralysed and Epileptic to determine the prognosis and curability of epilepsy. A total of 366 cases was used, and all were cases of genuine idiopathic epilepsy which had been under constant observation and treatment with bromides for a period of at least two years. All cases of symptomatic epilepsy or cases otherwise complicated were eliminated. The cases were divided into three series, according as they responded, successfully or otherwise to treatment—arrested, improved, and confirmed cases. A family history was found more frequently amongst those who became confirmed epileptics, but in any given case a hereditary history of epilepsy does not militate against the prospects of arrest or improvement. The age at the onset of the disease has an especial bearing on the prognosis. The most unsatisfactory cases are those in which the disease commences under ten years of age; they show the smallest percentage of recoveries and the largest of confirmed cases. If the disease arises between 15 and 20 years of age an almost equal percentage of arrested and confirmed cases may be expected. From that time onwards there is a steady increase in the expectation of arrest and diminution in the number which become confirmed. The duration of the malady influences the prognosis to the extent that arrest or improvement is much more likely during the first five than during the second five years. The frequency of the attacks is of great importance; the highest percentage of arrest occurs in those whose fits are as infrequent as once or twice a year. Major attacks are the most tractable, then follow combined major and minor seizures, and lastly the minor attacks occurring alone. Marriage exerts little if any influence upon epileptic fits. Pregnancy has little influence upon the seizures, whilst the puerperium is especially favourable for the recurrence of fits. If a period of remission for at least nine years be fixed as the basis upon which a cure may be established, 10·2 per cent. of cases are curable. In the cases in which arrest took place cessation of the fits occurred within the first year of continuous treatment in over 50 per cent.

**Syphilitic Disease of the Nervous System.**—In a lecture by Gowers[2] there are some practical hints on the treatment of syphilitic disease of the nervous system. It is probable that mercury has a capacity for arresting many diseases that depend on an organismal virus, although it is only in a slow disease that we can avail ourselves of this effect. Thus an animal under the influence of mercury is unaffected by a dose of anthrax virus which is fatal to an animal not so protected. On the other hand iodide does not affect any other organismal disease. For the chief lesions of constitutional syphilis they seem equally useful. But when the process is inflammation we should expect mercury to have the more certain influence, and there is a consensus of opinion that in the early stages of the disease mercury is more useful. Regarding the later processes there is a general feeling that mercury can do a little more and can do it more thoroughly than iodide, but that the latter does its work more speedily; but this does not rest on good evidence. And it is certain that the symptoms of a syphilitic growth may diminish as rapidly if iodide be given as with mercury. It is not wise to give iodide and mercury together in full doses except for a short time in very urgent cases, for there is reason to think that iodide promotes the elimination of mercury as it does of most metallic substances. Mercury should be given in doses sufficient to cause slight inflammation of the gums, otherwise we cannot be sure that there is enough in the blood to act on the tissue processes. The best plan is to rub the mercury into the skin, by this the gums can be affected in five or six days, which is as speedily as the result can be obtained by any system. Oleate of mercury (10 per cent.) is the most convenient preparation to use; a drachm of this should be rubbed in twice a day for three or four days, and then once a day until the end of a week. By this time the gums are generally affected; afterwards it may be applied at longer intervals. This specific treatment should be energetic, brief, renewed, but not continuous. It should stop at the end of eight weeks or so, be renewed after four or six months, and the patient should have three or four weeks' treatment with iodide every year for three or four years. One reason is that treatment may lose its power if continuously maintained; a second is that treatment has no influence on the latent germs of the disease, though destroying the developed and developing organisms, and thus dissipating the morbid processes they excite. Thus there may be recurrence after recurrence in some cases in spite of most thorough treatment.

[1] Lancet, June 13, 1903. [2] Brit. Med. Jour., April 4, 1903.

*(To be continued.)*

